# A Rare Case of Pseudomembranous Colitis Presenting with Pleural Effusion and Ascites with Literature Review

**DOI:** 10.1155/2021/6019068

**Published:** 2021-12-31

**Authors:** Hossain Salehi, Amir Mohammad Salehi

**Affiliations:** ^1^Gastroenterology Ward, Baharlo Hospital, Tehran University of Medical Sciences, Tehran, Iran; ^2^Medical Student, School of Medicine, Hamadan University of Medical Sciences, Hamadan, Iran

## Abstract

*Clostridium difficile* infection usually results from long-term and irregular antibiotic intake. The high-risk individuals for this infection include the patients undergoing chemotherapy due to malignancy, immunocompromised patients, and hospitalized patients receiving broad-spectrum antibiotics. The most common clinical manifestation of *Clostridium difficile* infection is diarrhea. However, pleural effusion and ascites have rarely been observed. As mentioned, these manifestations can be developed in a patient being treated with broad-spectrum antibiotics. Therefore, the present study reports a rare case of *Clostridium difficile* infection manifesting with these rare manifestations who was a 78-year-old female patient with a history of COVID-19, orthopedic surgery, and antibiotic treatment with cefixime and gentamicin.

## 1. Introduction

*Clostridium difficile* infection (CDI) is a major public health issue. Despite the remarkable changes in its diagnosis and treatment in recent years, the incidence of this infection has increased in hospitalized patients, as well as the community [1]. *Clostridium difficile* is a Gram-positive bacillus and obligate anaerobe whose spores abundantly occurred in nature. These spores can easily be swallowed by humans and proliferate in the distal parts of the intestine. This organism can initiate the destruction of the epithelial cell barrier and create a pseudomembrane by secreting toxins A and B [2]. The main risk factor for *Clostridium difficile* infection is a recent antibiotic exposure [3], and the infection is often neglected due to the lack of sensitive diagnostic tests and most importantly the lack of clinical suspicion. Although diarrhea is the most common clinical manifestation of CDI, the related manifestations varied and include hypovolemia, electrolyte imbalance, hypoproteinemia, toxic megacolon, gastrointestinal perforation, diffuse intravascular coagulation (DIC), and sepsis [4]. However, hydrothorax and ascites are very rare manifestations of CDI.

## 2. Case Report

A 78-year-old woman presented to the Emergency Department of the Baharloo Hospital with hematemesis since two days before presentation. The patient reported a history of COVID-19 contraction about one month ago with decreased oxygen saturation, followed by an orthopedic surgery due to a femoral neck fracture caused by falling. Moreover, she reported postoperative treatment with cefixime and gentamicin for one week and had received favipiravir, corticosteroids, and aspirin for 20 days due to her COVID-19 infection. Also, she did not report any history of underlying diseases or smoking. In physical examination, the patient had normal respiratory rate, heart rate, and body temperature. She had no abdominal tenderness; however, she looked ill.

Afterward, the patient underwent an endoscopy and was diagnosed with mucosal tearing in the gastroesophageal junction (Mallory Weiss syndrome). Therefore, local injection of epinephrine (1 : 10,000), followed by argon plasma coagulation (APC), was performed for the patient to maintain the hemostasis. The patient was then transferred to the gastroenterology and hepatology ward. The day following the endoscopy, she complained of abdominal pain, diarrhea, and dyspnea. In physical examination, she had a temperature of 37.8°C, a heart rate of 85 per minute, and a respiratory rate of 22 per minute. There was no erythema in the pharyngeal examination, and there was no cervical lymphadenopathy. In addition, heart sounds were normal, while there were decreased pulmonary sounds at the base of the lungs. Also, the patient's abdomen was distended and tender and had shifting dullness in percussion.

**Figure 1:**
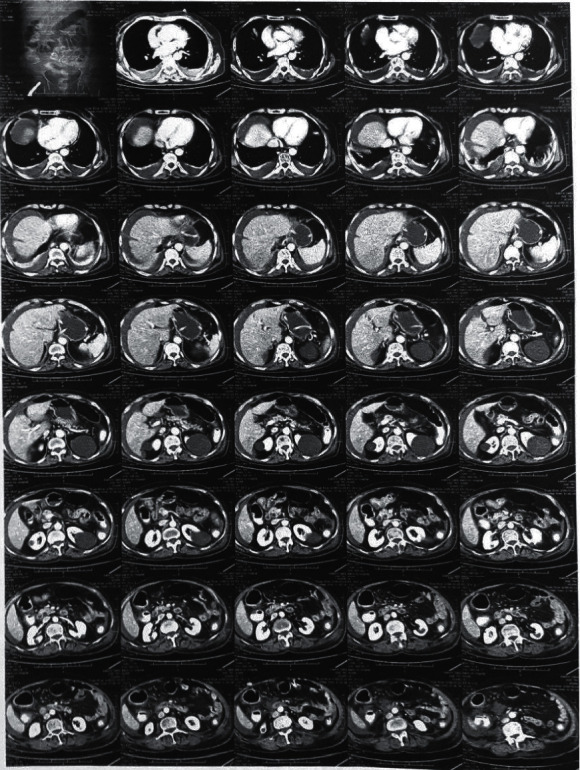
Computed tomography (CT) showing the presence of ascites and pleural effusion.

**Figure 2:**
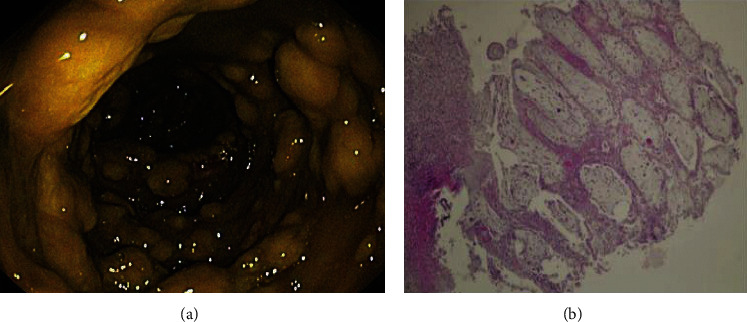
Patient colonoscopic findings (a). The presence of mucopurulent exudates (pseudomembrane) in pathological examinations indicates CDI (b).

Laboratory tests performed on the admission day showed leukocytosis (10400 per mm^3^), elevated CRP levels (220 mg/L), hypoalbuminemia (2.39 gr/dl), and elevated lactate dehydrogenase levels (511 U/L). First, an abdominal ultrasound was requested for the patient, which reported ascites. Then, the patient underwent a pulmonary CT scan, which confirmed pleural effusion (Figure 1). The patient underwent thoracocentesis and paracentesis, and the sample analysis showed an exudative pleural effusion and low-SAAG ascites. In addition, cytological examination and culturing of the ascites sample did not show any malignancies or bacterial, mycobacterial, and fungal infection (Table 1). Also, the patient underwent bronchoscopy and bronchoalveolar lavage (BAL) after consultation with a pulmonology specialist, showing unremarkable results. Therefore, the underlying cause of ascites and pleural effusion was not still diagnosed. Finally, the patient underwent abdominal CT scan for her abdominal pain, tenderness, and distension. The CT scan report suggested the possibility of colorectal cancer. Therefore, the patient underwent colonoscopy. She had yellow plaques throughout the colon, which underwent a biopsy. Finally, histological examinations suggested CDI (Figures 2(a) and 2(b)). Thus, the patient was treated with metronidazole (500 mg TDS) and vancomycin (125 mg every 6 h) for 14 days, leading to gradual improvement of her symptoms and resolution of ascites and pleural effusion.

## 3. Discussion

*Clostridium difficile* infection is the leading cause of hospital-acquired diarrhea in developed countries. However, it is usually underdiagnosed, most importantly due to lack of clinical suspicion [5].

The main risk factor for this infection is antibiotic exposure, especially with clindamycin, ampicillin, and cephalosporins. The second and third-generation cephalosporins, especially cefotaxime, ceftriaxone, cefuroxime, and ceftazidime, are commonly associated with this infection [2]. Furthermore, prolonged treatment with proton pump inhibitors (PPIs) can lead to intestinal infections, including CDI. Therefore, the patients undergoing long-term PPI treatment should be evaluated for CDI if they develop diarrhea [6]. In addition to antibiotic exposure, other risk factors for CDI include old age (older than 55), recent hospitalization, the history of CDI, malignancy, CRF, use of immunosuppressive drugs, and ICU admission [7].

The CDI diagnosis is based on a combination of clinical (passing watery stool 3 times a day for more than 2 days without other explanations) and paraclinical (detection of toxins A or B, toxicogenic *Clostridium difficile* detection using PCR, or pseudomembrane observation using colonoscopy) findings [2].

Most cases of CDI infections primarily presented with hydrothorax or ascites have been reported in immunocompromised adults (8 out of 14 cases reported), and there are rare reports in children (2 out of 14 cases reported) or immunocompetent adults (4 out of 14 cases reported) [4]. Up to now, 14 cases of CDI with ascites or hydrothorax as clinical manifestations have been reported, as given in Table 2. From these 14 patients, only 6 patients developed ascites and pleural effusion simultaneously. The mechanism of pleura effusion and ascites due to CDI remains unclear; however, hypoalbuminemia, colonic inflammation with microperforation and infectious peritonitis, and toxin-mediated cytokines enhancing vascular permeability could be involved [4, 8].

Since there is no effective vaccination against *Clostridium difficile*, several strategies have been emphasized to prevent the spread of the infection, such as antibiotic use limitations, contamination prevention of sanitary equipment and facilities, and use of probiotics [9]. Minimizing the use of antibiotics in hospitalized patients can effectively reduce the incidence of *Clostridium difficile* infection. For example, restrictions on ceftriaxone and ciprofloxacin administration combined with related instructions could reduce the infection rate by 77% in a hospital with 952 beds in Scotland [9]. Also, probiotic bacteria can replace the natural flora of the gastrointestinal tract, thus preventing diarrhea induced by *Clostridium difficile*. However, the evidence on the effectiveness of probiotics in CDI prevention is weak, despite the numerous clinical studies performed on this topic [10]. A large study investigating the effects of high-dose probiotic administration did not find clear evidence of the effectiveness of these drugs in CDI prevention. Therefore, the effective role of these agents has not yet been confirmed [10]. However, a systematic review including 38 randomized controlled trials on a total of more than 8000 patients showed that the use of the yeasts in the genus *Brassica* could regulate the intestinal flora and exert antitoxic effects. In addition, it has higher therapeutic effects on the CDI compared to other probiotics [11].

Since 1970, metronidazole and vancomycin have been introduced as the medications of choice for CDI treatment. Moreover, no clinically significant resistance to any of these drugs has been observed despite their administration for millions of patients [9]. Vancomycin is preferred in severe infections; however, these two are not significantly different in treating mild to moderate infections [9]. In 2011, fidaxomicin was approved by the Food and Drug Administration (FDA) of the United States for CDI treatment. The clinical studies performed showed the almost equal effectiveness of fidaxomicin and vancomycin in acute CDI treatment. Moreover, the recurrence risk was reportedly lower in patients treated with fidaxomicin [9]. Recently, fecal microbial transplant (FMT) has been introduced as the most recent therapeutic strategy for CDI, which is performed more than 3 times a day in the form of enema for patients with recurrent CDI. However, to treat the hydrothorax and ascites induced by CDI, the underlying disease should be treated, and the fluid should be drained if necessary [4].

## Figures and Tables

**Table 1:** The analysis of pleural and peritoneal fluid samples.

Factor	Ascites	Pleural effusion
Color	Yellow	Yellow
Appearance	Turbid	Semiclear
Leukocyte count (per mm^3^)	1312	400
Neutrophil (%)	80%	70%
Lymphocyte (%)	20%	30%
Glucose	125 mg/dl	81 mg/dl
Protein	3140 mg/dl	2100 mg/dl
RBC	610 mm^3^	250 mm^3^
Albumin	1900 mg/dl	1300 mg/dl
LDH	771 IU/L	344 IU/L
Ratio	SAAG: 0/49 (low SAAG)	Pleural LDH/serum LDH: 0.67 (exudative)

**Table 2:** Summary of case reports on CDI presenting with hydrothorax and ascites as the main symptoms.

Study by	Year	Age (y)	Primary disease	Antibiotics treatment before CDI/duration	Pleural effusion	Ascites	Antibiotic/duration	Outcome
Shen et al. [12]	2009	61	Ulcerative colitis/10 d after intestinal operation	Ampicillin IV/NG	−	+	Vancomycin PO/NG metronidazole IV/NG	Died
Dong in nam et al. [13]	2015	80	Hypertension	Ceftriaxone 2.0 g/day	+	+		Cured
Tsourous et al. [14]	2007	60	Type 2 diabetes/PAOD/soft tissue infection combined with osteomyelitis	Amoxicillin, clindamycin PO/40 d	−	+	Vancomycin PO/NG	Cured
Boaz et al. [15]	2000	25	Oral cavity infection	Clindamycin PO/10 d	+	+	Vancomycin PO/NG	Cured
Pang et al. [4] (according to the narration)	2015	71	Gastrointestinal bleeding due to cirrhosis	Ceftriaxone IV/14 d	−	+	Ornidazole PO/14 d	Cured
Zukerman et al. [16]	1997	54	AIDS/pneumocystosis	SMX, RMP PO/7 m	−	+	–	Died
		48	Heroin IV usage/URI	Erythromycin NG/7d	−	+	Metronidazole NG/NG	Cured
		30	AIDS/*P. aeruginosa* pneumonia	Ceftazidime, gentamicin IV/NG	−	+	Vancomycin PO/14 d	Cured
		33	AIDS	SMX, fluconazole NG/NG	−	+	Metronidazole/PO/14 d	Cured
		58	CAP	Erythromycin NG/6 d	−	+	Metronidazole PO/21 d	Cured
Zwiener et al. [17]	1982	2.5	Otitis media	Amoxicillin NG/10 d	+	+	Vancomycin PO/14 d	Relapsed
Yujian Liang et al. [4]	2020	6	Diffuse large B cell lymphoma	Vancomycin, imipenem IV/3 d	+	+	Vancomycin PO/12 d	Cured
Alsultan MH et al. [18]	2021	25	End-stage renal disease (ESRD)	Levofloxacin, ceftazidime, metronidazole	+	+	vancomycin (250 mg/4 times daily) metronidazole (500 mg/3 times daily)	Cured
Our case	2021	72	COVID-19	Cefixime, gentamicin	+	+	Vancomycin PO/14 d metronidazole/PO/14d	Cured

## Data Availability

The access to data used to support this study is permitted with the author's permission.
